# Perceived age discrimination and life satisfaction among older adults in China: mediating roles of leisure and social satisfaction

**DOI:** 10.3389/fpsyg.2026.1744251

**Published:** 2026-06-19

**Authors:** Rongwang Guo, Xiaoyan Li

**Affiliations:** 1 School of Agricultural Economics and Management, Shanxi Agricultural University, Jinzhong, China; 2 School of Management and Engineering, Shanxi University of Finance and Economics, Taiyuan, China

**Keywords:** age discrimination, leisure satisfaction, life satisfaction, older adults, social satisfaction

## Abstract

**Introduction:**

Rapid population aging has made age discrimination against older adults increasingly prominent. However, empirical evidence on how age discrimination affects life satisfaction among Chinese older adults, and through which mechanisms, remains limited.

**Methods:**

Using data from the 2021 China Social Survey (CSS), this study analyzed a sample of 840 older adults aged 60 and above. Ordinary least squares (OLS) regression and mediation analysis were employed to examine the direct effect of perceived age discrimination on life satisfaction, the mediating roles of leisure satisfaction and social satisfaction, and heterogeneity across gender and hukou (household registration) types.

**Results:**

Greater perceived age discrimination was significantly associated with lower life satisfaction. Both leisure satisfaction and social satisfaction mediated this relationship. The negative effect of age discrimination on life satisfaction was significantly stronger among older adults with rural hukou compared with their non-rural counterparts, whereas no significant gender-based differences were observed.

**Conclusion:**

This study extends the understanding of age discrimination's detrimental impact to the Chinese context, identifies leisure and social satisfaction as key mediators, and reveals rural-urban heterogeneity. These findings inform targeted interventions to reduce ageism and address structural inequalities, thereby promoting healthy aging in China and other rapidly aging societies.

## 1 Introduction

Population aging has emerged as a significant global challenge, prompting increased attention from researchers and policymakers to the needs of older adults. China, with one of the fastest aging populations in the world, is particularly affected. Current statistics from China's National Bureau of Statistics (2024) indicate that 310 million citizens aged ≥60 years constitute 22.0% of the total population, with the ≥65 cohort reaching 220 million (15.6%). In this study, older adults are defined as individuals aged 60 years and above, a threshold commonly adopted in aging research and aligned with the United Nations' definition of the older population (United Nations, Department of Economic and Social Affairs, Population Division, 2019). In response, the Chinese government has successively issued policies such as the Opinions of the CPC Central Committee and the State Council on Strengthening Gerontological Work in the New Era, the 14th 5-Year Plan for Healthy Aging, and the Opinions of the General Office of the State Council on Developing the Silver Economy to Enhance the Wellbeing of Older Adults. These initiatives aim to promote healthy aging and improve the life satisfaction and happiness of older adults. Life satisfaction constitutes a cognitive appraisal of one's overall quality of life according to self-perceived standards (Heller et al., 2004). The lives of older adults are primarily characterized by participation in leisure activities and social interactions, which are closely related to their life satisfaction and overall wellbeing (Michèle et al., 2019; Robertson et al., 2023; Yueh and Chang, 2024). Leisure satisfaction reflects the enjoyment derived from engaging in recreational activities, while social satisfaction indicates the fulfillment obtained from social interactions and relationships.

Contemporary evidence identifies age discrimination as an escalating worldwide challenge (Burnes et al., 2019; Levy et al., 2022). A recent UN report indicates that half of the world's population holds discriminatory attitudes toward older adults, leading to deteriorated physical and mental health, reduced quality of life, and substantial economic losses (World Health Organization, 2021).

Age discrimination, also termed ageism, refers to the stereotyping, prejudice, and discrimination directed toward individuals or groups based on their age (Palmore, 1989; World Health Organization, 2021). This multidimensional construct operates at three interrelated levels. Firstly, stereotypes encompass cognitive beliefs and expectations about older adults, such as assumptions that they are less competent, less flexible, or technologically incapable. Secondly, prejudice involves affective responses, including fear, disdain, or pity toward older individuals. Thirdly, discrimination manifests as behavioral actions, ranging from subtle exclusion in social interactions to overt denial of employment, healthcare, or housing opportunities. In the context of older adults specifically, age discrimination manifests through stereotypes and assumptions that they possess reduced capabilities, lack flexibility, are incapable of self-care, and constitute a burden to society (Levy et al., 2022; Sinclair et al., 2024). These discriminatory attitudes and behaviors are not merely abstract social phenomena but are embedded in everyday interactions, institutional practices, and cultural narratives that shape older adults' lived experiences.

Such discrimination profoundly undermines wellbeing, with recent studies specifically linking perceived ageism to lower life satisfaction, increased loneliness, and more depressive symptoms among older populations (Detwiler et al., 2023; Hajek and König, 2024). Given these significant negative impacts, addressing age discrimination is crucial, since reducing such experiences can directly enhance life satisfaction for older adults in China by alleviating the psychological and social harms that erode their quality of life. Across various countries and cultural contexts worldwide, aging is frequently linked to negative perceptions (Ayalon and Tesch-Römer, 2018; Levy and Apriceno, 2019; Swift et al., 2019).

A substantive body of literature confirms the deleterious consequences of ageism for multidimensional health in aging populations. A recent meta-analysis spanning 11 health domains established that 95.5% of included studies demonstrated consistent causal links between age discrimination and health deterioration (Levy et al., 2022). Specifically, age discrimination correlates with elevated disease burden and diminished longevity (Hu et al., 2021; Levy et al., 2022). It substantially compromises older adults' psychological wellbeing, exacerbating perceptions of social isolation and loneliness that predispose to clinical depression and anxiety disorders (Narita et al., 2023; Marchiondo et al., 2019). Beyond its psychological impact, age discrimination has been linked to higher incidence rates of various chronic conditions among workers, including hypertension, cardiovascular events, coronary heart disease, chronic pulmonary disease, arthritis, and other long-term restrictive illnesses (Levy, 2009; Jackson et al., 2019). Furthermore, age discrimination has been shown to exert negative effects on job satisfaction, motivation, and overall happiness among older adults (Marchiondo et al., 2019; Kornadt et al., 2020).

Surprisingly, given the rapid aging of China's population, few studies have specifically examined age discrimination among older adults in China, and the relationship between age discrimination and life satisfaction among this demographic remains unclear.

To address this research gap, this study poses the following research questions:

(1) Does perceived age discrimination negatively affect life satisfaction among Chinese older adults?
(2) Do leisure satisfaction and social engagement satisfaction mediate the relationship between age discrimination and life satisfaction?
(3) How does the impact of age discrimination on life satisfaction differ across gender and household registration (hukou) types?

To answer these questions, this study utilizes nationally representative data from the 2021 China Social Survey (CSS). Consistent with the definition established above, the analysis focuses on Chinese adults aged 60 and older, a group particularly vulnerable to the psychosocial harms of discriminatory attitudes. Specifically, this study first examines the direct impact of ageism on life satisfaction among older adults, then investigates the mediating roles of leisure and social engagement satisfaction, and finally conducts heterogeneity analyses to assess differential effects across gender and household registration (hukou) types.

The contributions of this study are threefold. First, by empirically validating the adverse effect of ageism on life satisfaction among Chinese older adults, the study extends extant theoretical frameworks to non-Western settings. Second, it identifies leisure satisfaction and social engagement as mediating mechanisms through which age discrimination affects life satisfaction. Third, it reveals heterogeneous effects across gender and hukou types, providing nuanced insights into how the impact of age discrimination varies across population subgroups. Together, these findings offer actionable evidence and policy-relevant insights for China and other rapidly aging societies to combat the detrimental impacts of ageism on older adults' life satisfaction through targeted interventions.

## 2 Materials and methods

### 2.1 Sample

This study employs data from the 2021 Chinese Social Survey (CSS), a longitudinal initiative established in 2005 by the Chinese Academy of Social Sciences' Institute of Sociology. The CSS aims to carry out extensive, ongoing assessments of labor employment, family and social life, and social attitudes among the general population across China, thereby furnishing nuanced and empirical data for social science research. Conducted biennially, the survey employs a probability sampling approach for face-to-face household interviews, ensuring respondent anonymity throughout the process.

The CSS2021 marks the eighth iteration of the survey, with a thematic focus on “Social Quality and Modernization.” It encompasses modules on social and political participation, which are pertinent to the objectives of this study. The CSS2021 successfully conducted household surveys in 592 villages and communities spanning 30 provinces (excluding Xinjiang province), municipalities, and autonomous regions in China, yielding a final analytical sample of 10,136. Following the definition established in the introduction, respondents under 60 years of age and those with missing data for key variables were excluded, resulting in a final sample of 840 respondents.

### 2.2 Statistical analyses

All statistical analyses were performed with STATA Version 17.0. The association of age discrimination with life satisfaction was modeled using OLS regression, investigating a nationally representative sample of older adults in China. To elucidate the underlying mechanisms, the mediating pathways through leisure satisfaction and social satisfaction were tested using a standard three-step approach for the association between age discrimination and life satisfaction. Following the established three-step procedure for mediation testing (Baron and Kenny, 1986), this study first regressed life satisfaction on age discrimination to establish the total effect. Next, the two proposed mediators (leisure and social satisfaction) were regressed separately on age discrimination. Finally, life satisfaction was regressed on both age discrimination and the mediators concurrently to estimate the direct effect and the mediation effects.

Furthermore, the significance of the indirect effects was tested using the bootstrap method, a non-parametric resampling technique that generates robust estimates by empirically constructing the sampling distribution, thereby yielding accurate inference even when normality assumptions are violated.

Additionally, the study tested for effect heterogeneity by estimating the model separately for each subgroup (defined by gender and hukou) and then comparing the coefficient for age discrimination across these groups. This analysis helps to identify whether the effect of age discrimination varies across subgroups defined by gender or household registration status, with findings that can inform targeted policy interventions.

#### Measurement

#### 2.3.1 Dependent variable

The measure for life satisfaction was a single-item question: “Overall, how satisfied are you with your life?” Responses were collected on a 10-point scale from 1 (very dissatisfied) to 10 (very satisfied), with scores being interpreted directionally (higher = more satisfied). Single-item self-assessment measures have been increasingly employed in recent studies to evaluate individual life satisfaction (Zhang et al., 2021; Delhey et al., 2023).

#### 2.3.2 Independent variable

Age discrimination was measured using a single-item question: “Do you perceive age-related unfair treatment in today's society to be a severe issue?” Responses were rated on a 4-point Likert scale ranging from 1 (very serious) to 4 (does not occur), with higher scores indicating lower perceived age discrimination. Single-item self-report measures of perceived discrimination have been widely employed and validated in large-scale survey research, demonstrating acceptable reliability and predictive validity (Roscigno et al., 2022; Guo et al., 2024).

#### 2.3.3 Mediating variables

Leisure satisfaction was assessed using a single-item self-report scale, following the methodological framework proposed by London et al. (1977). This was assessed with the question: “How satisfied are you with your leisure, entertainment, and cultural activities?” Participants indicated their level of satisfaction on a 10-point scale ranging between 1 (very dissatisfied) to 10 (very satisfied).

Social satisfaction was evaluated using the following question: “How satisfied are you with your social life?” Responses were recorded on a 10-point scale anchored by 1 (very dissatisfied) and 10 (very satisfied), where higher values corresponded to greater social satisfaction.

#### 2.3.4 Control variables

Covariates consisted of gender, age, marital status, educational level, hukou type, political status, ethnicity, and personal annual income, drawing on prior research (Zhang et al., 2021; Khodabakhsh, 2022).

## 3 Results

### 3.1 Descriptive results

This study first employs the Ordinary Least Squares (OLS) model for the baseline regression. Subsequently, a mediation analysis is conducted to examine the mediating effects.

Table 1 presents the characteristics of the variables utilized in this study. In the sample, approximately 49.64% of older adults were male, with a mean age of 64.852 years (range: 60–70 years). The educational level was relatively low, with 46.66% of older adults having completed primary school or less. Most participants (86.07%) were married or in a spousal relationship, and the sample was predominantly Han Chinese (95.24%). Furthermore, 64.52% of older adults held rural household registration, and 85.60% of them identified as members of the general public (non-party members).

**Table 1:** Descriptive statistics of the sample (*N* = 840).

Variables	Mean	SD	Min	Max
Life satisfaction	7.573	2.293	1	10
Leisure satisfaction	6.211	2.990	1	10
Social satisfaction	7.069	2.745	1	10
Age discrimination	2.868	1.022	1	4
Gender	1.504	0.500	1	2
Age	64.852	2.797	60	70
Education	1.299	0.555	1	3
Marital status	2.127	0.368	1	3
Hukou	1.355	0.479	1	2
Political status	3.574	1.042	1	4
Ethnic	1.048	0.213	1	2
Wage (Chinese Yuan, logarithm)	8.677	2.615	0	12.206
Family size	4.977	2.696	1	25

### 3.2 Benchmark regression

Table 2 presents the OLS results examining the impact of age discrimination on life satisfaction among older adults. Model 1 includes only age discrimination as a predictor, showing a significant positive coefficient (β = 0.539, *p* < 0.001). After adding sociodemographic covariates in Model 2, the coefficient slightly decreased but remained statistically significant (β = 0.522, *p* < 0.001). Because higher scores on the age discrimination measure indicate lower perceived discrimination, these positive coefficients indicate that lower perceived age discrimination is associated with higher life satisfaction. In other words, higher perceived age discrimination is associated with lower life satisfaction.

**Table 2:** Direct effects regression results.

Variables	Life satisfaction	
	Model 1	Model 2
Age discrimination	0.539^***^	0.522^***^
	(0.077)	(0.076)
Gender		0.346^**^
		(0.164)
Age		0.047
		(0.029)
Edu		−0.127
		(0.163)
Married		−0.543^**^
		(0.213)
Hukou		0.334^***^
		(0.110)
Political status		−0.111
		(0.082)
Ethnic		−0.206
		(0.418)
Wage		0.035
		(0.032)
Family size		0.008
		(0.031)
Region FE	YES	YES
_cons	6.498^***^	3.746^*^
	(0.517)	(2.158)
N	840	840
*R* ^2^	0.113	0.144

Standard errors are shown in parentheses,

^***^p < 0.001;

^**^p < 0.05;

^*^p < 0.10.

Several control variables also showed statistically significant associations. Female respondents reported higher life satisfaction than males (β = 0.346, *p* < 0.05). Married respondents reported lower life satisfaction than their unmarried counterparts (β = −0.543, *p* < 0.05). Hukou type was positively associated with life satisfaction (β = 0.334, *p* < 0.001), indicating higher satisfaction among urban residents. Other control variables (age, education, political status, ethnicity, wage, family size) were not statistically significant.

### 3.3 Mediation analysis results

#### 3.3.1 Three-step mediation analysis

Table 3 presents the results of the three-step mediation analysis examining the potential mediating roles of leisure satisfaction and social satisfaction in the relationship between age discrimination and life satisfaction.

**Table 3:** Mediation analysis results.

Variables	Leisure satisfaction	Social satisfaction	Life satisfaction	Life satisfaction
	Model 1	Model 2	Model 3	Model 4
Age discrimination	0.423^***^	0.312^***^	0.362^***^	0.384^***^
	(0.102)	(0.094)	(0.067)	(0.064)
Leisure satisfaction			0.380^***^	
			(0.023)	
Social satisfaction				0.443^***^
				(0.024)
Gender	0.318	0.536^**^	0.225	0.108
	(0.219)	(0.202)	(0.141)	(0.138)
Age	0.029	0.053	0.036	0.023
	(0.038)	(0.035)	(0.025)	(0.024)
Edu	0.143	−0.212	−0.181	−0.033
	(0.217)	(0.201)	(0.140)	(0.136)
Married	−0.176	−0.593^**^	−0.476^**^	−0.280
	(0.284)	(0.263)	(0.183)	(0.179)
Hukou	0.446^**^	−0.074	0.164^*^	0.366^***^
	(0.146)	(0.135)	(0.095)	(0.092)
Political status	−0.150	−0.043	−0.054	−0.092
	(0.109)	(0.101)	(0.071)	(0.068)
Ethnic	−0.464	−0.325	−0.029	−0.062
	(0.559)	(0.516)	(0.360)	(0.350)
Wage	0.083^*^	0.089^**^	0.003	−0.005
	(0.043)	(0.040)	(0.028)	(0.027)
Family size	−0.057	0.038	0.030	−0.009
	(0.041)	(0.038)	(0.027)	(0.026)
Region FE	YES	YES	YES	YES
_cons	2.602	3.615	2.756	2.144
	(2.885)	(2.664)	(1.861)	(1.810)
*N*	840	840	840	840
*R* ^2^	0.100	0.090	0.365	0.400

Standard errors are shown in parentheses,

^***^p < 0.001;

^**^p < 0.05;

^*^p < 0.10.

The regression results demonstrated that age discrimination was significantly and positively associated with both leisure satisfaction (Model 1: β = 0.423, *p* < 0.001) and social satisfaction (Model 2: β = 0.312, *p* < 0.001). In subsequent models, both mediators showed significant positive associations with life satisfaction (Model 3: leisure satisfaction β = 0.380, *p* < 0.001; Model 4: social satisfaction β = 0.443, *p* < 0.001). Importantly, the direct effect of age discrimination on life satisfaction remained statistically significant at the 1% level even after including the mediators.

Compared to the total effect of age discrimination on life satisfaction reported in Table 2 (β = 0.522, *p* < 0.001), the direct effect coefficient decreased upon the introduction of the two mediators. This reduction indicates that both leisure satisfaction and social satisfaction partially mediate the relationship between age discrimination and life satisfaction.

#### 3.3.2 Bootstrap test results

To further examine the reliability of the mediating effects, this study employed the Bootstrap sampling method, conducting 500 replications to investigate the mediating effects of leisure satisfaction and social satisfaction. The results are presented in the lower half of Table 4. The 95% confidence intervals for the mediating effects of both leisure satisfaction and social satisfaction do not include zero, indicating that both variables significantly mediate the relationship between age discrimination and life satisfaction among older adults. Specifically, the mediating effect of leisure satisfaction is 0.167 [95% CI: (0.082, 0.252)], and the mediating effect of social satisfaction is 0.342 [95% CI: (0.186, 0.498)]. These results indicate that leisure satisfaction and social satisfaction serve as significant partial mediators in the relationship between age discrimination and life satisfaction.

**Table 4:** Bootstrap mediation analysis results.

Effect	Coefficient	S.E.	*Z*	*P*	95% CI
*Mediator: leisure satisfaction*					
Direct effect	0.386	0.077	5.02	0	[0.235, 0.536]
Indirect effect	0.124	0.043	2.86	0.004	[0.039, 0.208]
*Mediator: social satisfaction*					
Direct effect	0.342	0.080	4.29	0	[0.186, 0.498]
Indirect effect	0.167	0.043	3.86	0	[0.082, 0.252]

Results are based on 500 bootstrap replications. All models include controls for the covariates presented in Table 1. S.E., standard error; CI, confidence interval.

#### 3.3.3 Heterogeneous analysis

Table 5 presents the heterogeneity analysis results of the impact of age discrimination on life satisfaction, stratified by gender and household registration type. Model 1 and Model 2 show the regression results for the effect of age discrimination on life satisfaction among male and female older groups, respectively. Model 3 and Model 4 present the regression results for the effect of age discrimination on life satisfaction among older groups with rural and non-rural household registration, respectively.

**Table 5:** Heterogeneous analysis results.

Variables	Male	Female	Rural	Non-Rural
	Model 1	Model 2	Model 3	Model 4
Age discrimination	0.545^***^	0.549^***^	0.646^***^	0.182
	(0.115)	(0.108)	(0.097)	(0.124)
Age	0.066	0.050	0.018	0.110^***^
	(0.042)	(0.042)	(0.039)	(0.042)
Edu	−0.229	0.112	−0.034	−0.113
	(0.228)	(0.242)	(0.310)	(0.180)
Married	−0.376	−0.664^**^	−0.467	−0.516^*^
	(0.320)	(0.295)	(0.294)	(0.302)
Hukou	0.199	0.406^***^		
	(0.165)	(0.152)		
Political status	−0.113	−0.116	−0.173	−0.025
	(0.097)	(0.170)	(0.125)	(0.103)
Ethnic	−0.469	0.087	−0.349	−0.778
	(0.641)	(0.565)	(0.553)	(0.667)
Wage	0.124^**^	−0.019	0.027	−0.017
	(0.059)	(0.039)	(0.041)	(0.054)
Family size	−0.057	0.060	0.000	0.035
	(0.047)	(0.042)	(0.041)	(0.045)
Gender			0.287	0.362
			(0.223)	(0.237)
_cons	2.125	4.032	5.595^*^	2.156
	(3.237)	(3.096)	(3.146)	(3.181)
*N*	417	423	542	298
*R* ^2^	0.190	0.185	0.186	0.176

Standard errors are shown in parentheses,

^***^p < 0.001;

^**^p < 0.05;

^*^p < 0.10.

The results indicate that, except for the non-rural group, the regression results for the other three subgroups are significant. Specifically, age discrimination significantly affects life satisfaction in both male (β = 0.545, *p* < 0.001) and female (β = 0.549, *p* < 0.001) older groups, suggesting no significant difference in the impact of age discrimination on life satisfaction between genders. However, the impact of age discrimination on life satisfaction is stronger among those with rural household registration (β = 0.646, *p* < 0.001) compared to those with non-rural household registration (β = 0.182, *p* = 0.156), indicating a significant heterogeneity by household registration type.

## 4 Discussion

Employing nationally representative data from the CSS2021, this study investigated the detrimental impact of age discrimination on life satisfaction among Chinese older adults, specifically examining the mediating mechanisms of leisure and social satisfaction and heterogeneity across gender and hukou type. The findings conclusively demonstrate that ageism significantly undermines life satisfaction in this population. This finding aligns with prior research demonstrating that age discrimination negatively affects various aspects of older adults' wellbeing, including physical and mental health, job satisfaction, and overall happiness (Kornadt et al., 2021; McConatha et al., 2022; Detwiler et al., 2023; Hajek and König, 2024). As a global crisis (Levy et al., 2022), age discrimination undoubtedly exerts a negative influence on China's labor market (Jia et al., 2022). Consequently, age discrimination adversely impacts the physical and mental health of Chinese older adults, thereby diminishing their life satisfaction. This negative association is pronounced across different cultural contexts, implying that the negative association is generalizable beyond specific demographic or geographic confines.

The findings identify leisure satisfaction and social satisfaction as critical mechanisms linking age discrimination to reduced life satisfaction. This study establishes that both leisure and social satisfaction play significant mediating roles in the pathway between age discrimination and diminished life satisfaction. The daily lives of older adults are primarily centered around leisure and social activities, which play an indispensable role in their wellbeing (Robertson et al., 2023; Yueh and Chang, 2024). A body of evidence indicates that physical and mental wellbeing in older adults can be enhanced through leisure activity satisfaction, while life satisfaction is improved by establishing social connections (Tarkar et al., 2023; Luo et al., 2024; Hancock et al., 2025). Therefore, upon being subjected to age discrimination, older adults are less likely to achieve satisfaction in their daily leisure and social activities, which also implies lower life satisfaction.

From the gender-stratified results, age discrimination had no significant differential impact on the life satisfaction of male and female older adults, indicating that both genders experience comparable reductions in life satisfaction due to age discrimination. However, the negative impact of age discrimination on life satisfaction is stronger for older adults with rural hukou than for their urban counterparts.

This disparity stems primarily from two factors, foremost among them China's unique household registration (hukou) system. Previous studies have shown that rural hukou is associated with lower life satisfaction among older adults (Appau et al., 2020; Zhang et al., 2021). Holders of rural hukou may experience both age discrimination and hukou discrimination, which can have a compounded negative impact on their mental health and life satisfaction (Ge et al., 2024). Second, regional differences may also contribute to this gap. Non-rural hukou holders, who typically reside in urban areas, have higher social security income and socioeconomic status (Yuan et al., 2023). Urban areas offer a more comprehensive policy support system, richer recreational resources, and more complete social interaction venues, which promote greater participation in these activities. Conversely, rural regions are frequently deficient in comparable resources and infrastructure, which restricts older adults' access to fulfilling leisure and social engagement (Cui et al., 2022). Therefore, despite facing age discrimination, non-agricultural hukou holders can still mitigate negative emotions and enhance wellbeing through participation in leisure and social activities, resulting in a smaller impact on life satisfaction (Hancock et al., 2025). As hukou system reforms progress, these differential impacts are expected to gradually diminish in the future. This finding aligns with (Ayalon and Roy, 2023), who argue that age discrimination in the Western Pacific is often intertwined with institutional inequalities such as rural-urban divides. This study provides empirical evidence for this regional perspective, showing that rural hukou status compounds the negative effect of ageism on life satisfaction.

This study has several limitations that should be acknowledged. First, the exclusive focus on older adults (aged 60 and above) in China, while providing valuable insights within this context, curtails the generalizability of the findings. Future research would benefit from cross-cultural comparisons to validate the impact of age discrimination across diverse societal contexts and to elucidate the role of cultural factors in shaping this relationship. Second, the investigation concentrated solely on age discrimination, without considering its potential intersection with other forms of prejudice. As highlighted by recent scholarship (e.g., Guo et al., 2024), an intersectionality framework is crucial. In China's labor market, discrimination based on gender, education, or other factors may interact synergistically with ageism, potentially exacerbating its adverse effects on older adults. Future studies should explore these complex synergistic pathways to gain a more holistic understanding of the compounded burdens faced by older individuals. Third, the scope of this analysis was restricted to the individual-level consequences of age discrimination. This micro-level focus, while valuable, precludes an examination of its broader macroeconomic ramifications. As underscored by Voss et al. (2018), age-based discrimination can impair aggregate labor market efficiency and economic productivity. Future studies should therefore integrate macro-level data to quantify the societal and economic costs of ageism, thereby providing a more comprehensive assessment of its full societal impact.

## 5 Conclusion

Based on a sample of 840 older adults aged 60 and above from the CSS2021 dataset, this study demonstrates that age discrimination exerts a direct negative effect on life satisfaction among older adults. Furthermore, it indirectly undermines life satisfaction by diminishing satisfaction with leisure and social activities. The adverse effect of age discrimination is more pronounced among rural older adults, whereas no significant gender-based differences were observed. Against the backdrop of the Chinese government's active promotion of healthy aging, addressing age discrimination, a pervasive issue worldwide, is of critical importance. This study highlights the detrimental impact of age discrimination on the quality of life of older adults in China and underscores the urgency of mitigating such discrimination to improve their wellbeing. The findings also provide valuable insights that may serve as a reference for other developing countries seeking to enhance the living conditions and welfare of their aging populations. Future research and policy efforts should prioritize the development of comprehensive strategies to counteract age discrimination and foster an inclusive society that recognizes and values the contributions of older adults.

In light of these results, the following recommendations are advanced:

First, comprehensive public awareness campaigns and educational initiatives should be launched to highlight the detrimental impacts of age discrimination on mental health and wellbeing. Beyond general messaging, these campaigns should specifically target ageist stereotypes embedded in everyday language and communication practices. For instance, media guidelines could be developed to discourage patronizing or infantilizing terms when referring to older adults, while training programs for healthcare providers, social workers, and customer service professionals could emphasize respectful and person-centered communication. Such linguistic and behavioral interventions are critical, as the ways in which older people are spoken to and about shape societal conceptualizations of aging and can either reinforce or challenge negative stereotypes (Levy, 2009). Shifting these discursive practices is fundamental to reducing discriminatory attitudes and fostering a culture of respect and inclusivity.

Second, community centers and local organizations should develop accessible and diverse programs to encourage older adults' participation in social and leisure activities. Given that both leisure and social satisfaction mediate the effect of age discrimination, promoting engagement in these areas can enhance wellbeing and buffer against the adverse effects of discrimination. To maximize effectiveness, these programs should be co-designed with older adults themselves, ensuring they reflect participants' interests, abilities, and cultural preferences. Examples include intergenerational book clubs, community gardening projects, digital literacy workshops tailored to older learners, and peer-led exercise groups. Such participatory approaches not only strengthen social connections but also challenge ageist assumptions about older adults' capabilities and interests.

Finally, policy efforts must prioritize narrowing the urban-rural gap in public eldercare services. The significant rural-urban disparity identified calls for targeted structural interventions, including increased investment in rural nursing homes and community service centers to match urban standards, subsidized transportation to facilitate rural older adults' access to urban healthcare and social facilities, and the establishment of mobile service teams to deliver recreational and health programs in remote villages. Additionally, further reform of the hukou system is essential to reduce institutional barriers that prevent rural migrants from accessing urban social services upon relocation, thereby alleviating the compounded disadvantages of age and hukou discrimination.

## Data Availability

Publicly available datasets were analyzed in this study. This data can be found here: its the CSS 2021 dataset used in this study is publicly available (http://css.cssn.cn/css_sy/).
